# Novel Sensitive Electrochemical Immunosensor Development for the Selective Detection of HopQ *H. pylori* Bacteria Biomarker

**DOI:** 10.3390/bios13050527

**Published:** 2023-05-08

**Authors:** Hussamaldeen Jaradat, Ammar Al-Hamry, Mohammed Ibbini, Najla Fourati, Olfa Kanoun

**Affiliations:** 1 Measurement and Sensor Technology, Chemnitz University of Technology, 09126 Chemnitz, Germany; hujar@hrz.tu-chemnitz.de (H.J.); ammar.al-hamry@etit.tu-chemnitz.de (A.A.-H.); 2 Department of Biomedical Engineering, Jordan University of Science and Technology, Irbid 22110, Jordan; mohib@just.edu.jo; 3 SATIE Laboratory, UMR CNRS 8029, Conservatoire National des Arts et Métiers, 75003 Paris, France; fourati@cnam.fr

**Keywords:** immunosensor, *H. pylori*, HopQ, saliva, biosensor, nanotechnology, CNT, biomedical engineering

## Abstract

Helicobacter pylori (*H. pylori*) is a highly contagious pathogenic bacterium that can cause gastrointestinal ulcers and may gradually lead to gastric cancer. *H. pylori* expresses the outer membrane HopQ protein at the earliest stages of infection. Therefore, HopQ is a highly reliable candidate as a biomarker for *H. pylori* detection in saliva samples. In this work, an *H. pylori* immunosensor is based on detecting HopQ as an *H. pylori* biomarker in saliva. The immunosensor was developed by surface modification of screen-printed carbon electrodes (SPCE) with MWCNT-COOH decorated with gold nanoparticles (AuNP) followed by HopQ capture antibody grafting on SPCE/MWCNT/AuNP surface using EDC/S-NHS chemistry. The sensor performance was investigated utilizing various methods, such as cyclic voltammetry (CV), electrochemical impedance spectroscopy (EIS), and scanning electron microscope (SEM) coupled with energy-dispersive X-ray spectroscopy (EDX). *H. pylori* detection performance in spiked saliva samples was evaluated by square wave voltammetry (SWV). The sensor is suitable for HopQ detection with excellent sensitivity and linearity in the 10 pg/mL–100 ng/mL range, with a 2.0 pg/mL limit of detection (LOD) and an 8.6 pg/mL limit of quantification (LOQ). The sensor was tested in saliva at 10 ng/mL, and recovery of 107.6% was obtained by SWV. From Hill’s model, the dissociation constant Kd for HopQ/HopQ antibody interaction is estimated to be 4.60 × 10^−10^ mg/mL. The fabricated platform shows high selectivity, good stability, reproducibility, and cost-effectiveness for *H. pylori* early detection due to the proper choice of biomarker, the nanocomposite material utilization to boost the SPCE electrical performance, and the intrinsic selectivity of the antibody–antigen approach. Additionally, we provide insight into possible future aspects that researchers are recommended to focus on.

## 1. Introduction

*Helicobacter pylori* (*H. pylori*) is a gram-negative pathogenic bacterium that is hosted by almost 50% of people worldwide [1,2]. According to the World Health Organization (WHO) and the International Agency for Research on Cancer (IARC), *H. pylori* is considered a class I carcinogen [3,4,5,6]. In 2005, B. Marshall and R. Warren received the Nobel Prize in Physiology or Medicine for the discovery of *H. pylori* bacteria in 1982 [7]. *H. pylori* infects the gastric mucosa layer of the human gastrointestinal (GI) tract and can endure in such a harsh environment for a lifetime [8,9]. *H. pylori* colonization causes gastric diseases that, synergically with its consequent host’s inflammatory response and dietary/lifestyle factors, can lead to cancer [10]. Eventually, *H. pylori* infection is a causative agent of chronic gastritis, ulcers, and gastric cancer that can lead to death [8,9,10]. Most *H. pylori* infections occur through bacteria transmission through individual–individual interaction or when in contact with contaminated mediums such as communal spaces or contaminated food and water. The exact routes for *H. pylori* transmission are still unproven, but saliva is one of the body fluids containing *H. pylori*, proving the oral–oral transmission [11,12,13].

Several conventional approaches are available for detecting *H. pylori* infection, including invasive and non-invasive techniques such as urea breath test, stool antigen test, serology, and biopsy. Still, these techniques experience several limitations, such as their complexity, the need for highly skilled staff, they are time-consuming, their high cost, and their limited shelf-life [14,15,16,17,18]. Some *H. pylori* tests, such as the urea breath test or the stool antigen test, detect active infection cases only. However, other techniques, such as serology, can indicate a former exposure to *H. pylori*. In addition, testing for children and incapable adult cases can be very challenging, especially for invasive or semi-invasive testing, such as biopsies and ^14^C-urea breath tests [16,19]. Thus, the advent of biosensors as an analytical tool in the clinical and environmental detection of virulent microbiomes that cause diseases plays an intriguingly important role. In particular, biosensors with biomarker-based detection can offer very high accuracy in a complex sample such as saliva [16,18].

Considering the biosensor platform, the immunosensors are fundamentally based on transducing the highly specific immunoreaction between antibodies (receiver) and their (partially) complementary antigen/protein/peptide/hapten (biomarker) [20,21,22]. Immunosensors based on electrochemical methods are inherently very sensitive to infinitesimal chemical events at the electrode surface. Therefore, electrochemical immunosensors are very sensitive, giving rise to a very low limit of detection (LOD), high sensitivity, specificity, and selectivity [21,23,24,25]. The electrochemical biosensor field has reached a milestone, driven by the highly enhanced sensitivity, excellent selectivity, lower detection limits, detection ranges, shelf-life, simplified sample preparation, and cost affordability [23,25,26,27,28]. The use of nanomaterials and the scaling down of electrode geometry in biosensors prove to overcome fundamental limitations imposed by classical methods, especially in terms of sensitivity and LOD [27,29]. Therefore, the biosensor’s inherent sensitivity is one of the main driving forces for biosensor development investigations [21,27,28].

Regarding the *H. pylori* recognition elements, antibodies are considered the gold standard for specific and selective biomarker recognition [15,21,25,27,30]. The choice of a biomarker is partially decided by the reliability of utilizing it as evidence for the existence of bacteria in humans regardless of the infection status if active or passive, as well as for contamination monitoring in food, water, or environmental mediums [16]. *H. pylori* utilizes its outer membrane HopQ proteins to facilitate the mechanism of transfer of its pathogenic factor, such as CagA, to the host cells at early stages of *H. pylori* infection [8,9,31,32]. Therefore, HopQ is considered an excellent biomarker for reliable, selective, and specific non-invasive direct detection of *H. pylori* bacteria existence regardless of the status of the infection [4,12,33,34,35,36,37,38,39]. Biomarkers detection in human saliva is very promising because it is non-invasive and convenient for infection testing in a wider and more diverse range of patients, including children [40]. In addition, monitoring *H. pylori* bacteria contamination of media such as water and food can prevent the spread of *H. pylori* [41]. The strategy of nominating a biomarker and targeting early infection stages is one step further toward fighting the spread of the contagious carcinogen *H. pylori*, which also results in curbing its antimicrobial resistance development [32,41].

Biomarker-based biosensors need to be one-time use (disposable), cost-affordable, simple, accurate, reproducible, and sensitive [42,43,44,45,46]. Screen-printed carbon electrodes (SPCEs) are famous for their simple designs, low cost, and mass production, making them suitable for developing disposable biosensing platforms. Electrochemical SPCEs are designed with a 3-electrode configuration, working electrode (WE), counter electrode (CE), and reference electrode (RE). The WE and CE are printed with graphitic carbon-based ink, while the RE is printed with Ag/AgCl or Ag/Cl inks. Electrochemical immunosensing relies on monitoring the charge transfer capabilities between the medium that contains the analyte under investigation and the WE surface. Therefore, the WE of SPCEs is usually modified with nanomaterials to upstream their electrical properties and introduce diverse surface functionalization for several applications [30,45,47,48,49].

Nanomaterials are increasingly used in electrochemical biosensors due to their unique physicochemical properties and high surface area-to-volume ratio. Electrochemical biosensors combined with nanotechnology allow various surface modifications that boost up and upscale the loading capacity of the sensing surface [21,23,24,27,28,50,51]. Amongst several nanomaterials, carbon-based nanomaterials have been broadly investigated and studied, driven by their unique properties such as high conductivity, mechanical stability, and biocompatibility. Carbon nanotubes (CNTs) are a highly distinctive class of nanostructured carbon materials, thanks largely to their unique 2D wrapped-like structure, which confers exceptional electronic ballistic transfer capabilities. CNTs are considered one-dimensional needle-looking hollow cylindrical graphitic carbon nanostructures with Sp^2^ atom arrangements. CNTs chirality has several arrangements, theoretically considered as a rolled-up graphene sheet(s) structure. Among zigzag and single-walled CNTs (SWCNTs), multi-walled CNTs (MWCNTs) have superior qualifications, such as simplicity in fabrication, low-cost mass production, chemical inertness, and stability [52,53,54,55]. MWCNTs are highly effective as surface-modifying materials for electrochemical sensing applications due to their ability to enhance electrical surface conductivity, which upstream sensor’s sensitivity. Their unique ability to form a network on an electrode’s surface and facilitate high electronic transfer make them particularly well-suited for this purpose. In addition, MWCNTs, due to their multi-cylindrical concentric structure, allow for different chemical functionalizations on the outer layers with groups such as the carboxyl group (MWCNT-COOH), while the inner cylinders preserve the electrical properties. The MWCNT-COOH offers the capability to chemically immobilize antibodies through amide bonding between the MWCNT carboxyl end and the antibody amino group, which is necessary for the covalent immobilization of HopQ antibodies on the electrode surface [52,53,56,57,58,59]. Amongst metallic nanomaterials, gold nanoparticles (AuNPs) are one of the most widely used metallic nanoparticles, especially for protein stabilization. AuNPs biocompatibility plays an important role in preserving the biological activity of antibodies and proteins [60]. Biomolecules’ weaker electrical conductivity hampers the electronic exchange between the redox couple and the electrode surface. Therefore, utilizing AuNPs greatly enhances surface conductivity, which increases the sensor’s sensitivity and enlarges the effective surface area available for charge transfer. In addition, AuNPs enhance antibody–antigen interaction, which upscale selectivity [48,49,60]. Herein, the focus is on harnessing the aforementioned outstanding properties of MWCNT-COOHs and AuNPs to realize an electrochemical-based biosensor to detect *H. pylori*’s HopQ protein.

A few electrochemical immunosensors have been developed to detect *H. pylori* infection in humans based on virulent biomarkers, mostly in stool or blood samples [16,34,41]. However, *H. pylori* transmission routes are still elusive; for example, humans can become infected by using contaminated water and food, interacting with people, and using contaminated tools and facilities [16]. Hence, it is anticipated that endeavors toward detecting *H. pylori* will concentrate on clinically simplifying the test procedure and increasing the reliability of biosensors in less invasive mediums, such as saliva, to curb its spread. In addition, simple *H. pylori* tests are encouraged to broaden the range of environmental samples, such as water or food, to help fight the spread of bacteria. Researchers reported electrochemical immunosensors for *H. pylori* detection based on antibody–antigen interaction for human infection [61,62,63,64,65]. All the utilized biomarkers are virulent factors the bacteria inject into infected tissue(s) after infection [10]. However, the strategy of nominating the HopQ protein as a biomarker is novel due to its role in facilitating *H. pylori* adhesion to the GI epithelial tissue and due to its existence on the bacteria’s outer membrane [8,9]. Therefore, it is more advantageous than other biomarkers involved in later stages of infection. Consequently, utilizing this protein as a biomarker offers distinct advantages over other biomarkers. Thus, there is still a crucial need for *H. pylori* sensors to detect infections in humans at earlier stages and to monitor and track the bacteria in various environmental samples such as water supplies, fruits, and vegetables [16,17,18,24,66,67].

The primary objective of this study is to design and implement a simple and affordable immunosensor based on SPCEs using a novel biomarker selection strategy. The biosensor developed in this study is tested for its ability to detect *H. pylori* through the analysis of saliva samples, making it minimally invasive and suitable for a wider range of patients, including children. This unique feature enables convenient, more non-invasive, and time-efficient early-stage detection of *H. pylori* within clinical visit time. Several characterizations are performed to optimize, confirm, and realize the sensing platform, such as cyclic voltammetry (CV), electrochemical impedance spectroscopy (EIS), square wave voltammetry (SWV), energy dispersive spectroscopy (EDX), and scanning electron microscopy (SEM). Various performance analytical studies were carried out, such as reproducibility, selectivity, shelf-life, cross-reactivity, and recovery study in artificial saliva samples. To the best of our knowledge, this work is the first to report the utilization of HopQ as a biomarker for *H. pylori* in an electrochemical immunosensor.

## 2. Materials and Methods

### 2.1. Apparatus

Electrochemical characterization/pretreatment was performed using palmsens4 potentiostat purchased from PalmSens BV (GA Houten, The Netherlands). Scanning electron microscopy (SEM) was performed using an FEI Nova NanoSEM 200 microscope. Ultrasonic dispersion was performed using a BANDELIN SONOPULS mini20 homogenizer (Bandelin, Berlin, Germany). The Socorex Acura^®^ XS 826 precision Micropipettes 0.5–10 μL was purchased from Socorex Isba SA (Ecublens, Switzerland). Polyester-substrate-based SPCEs (ItalSens IS-C) were purchased from PalmSens BV (GA Houten, The Netherlands). All graphs were produced using OriginPro, Version 2021b, obtained from OriginLab Corporation (Northampton, MA, USA).

### 2.2. Reagents

The HopQ protein and its antibody were purchased from Biotrend Chemikalien GmbH (Köln, Germany). The HopQ protein is especially sequenced by Biotrend company for this experiment. MES acid was obtained from Thermo Fisher GmbH (Kandel, Germany). All other chemical reagents were obtained from Sigma-Aldrich Chemie GmbH (Taufkirchen, Germany), including artificial saliva, 2-Mercaptoethanol, Sulfo-N-Hydroxysulfosuccinimide sodium salt (S-NHS), Gold (III) chloride tri-hydrate (HAuCl_4_·3H_2_O), tri-sodium citrate dehydrate, N-(3-Dimethylaminopropyl)-N′-ethylcarbodiimide hydrochloride (EDC) 98%, disodium hydrogen phosphate, potassium dihydrogen phosphate, phosphate buffer saline (PBS), K_3_Fe(CN)_6_, K_4_Fe(CN)_6_, potassium chloride, MWCNTs, polyethyleneimine (Mn 60,000 g/mol) and gold standard solution for inductively coupled plasma (ICP) of (1000 mg/L Au), and Bovine serum albumin (BSA), all without any further purification or treatment.

### 2.3. Immunosensor Preparation

#### 2.3.1. Activation and Pretreatment of SPCE

The bare SPEC was pretreated with MES buffer (0.01 M) electroactively with CV from −1–+1 V at a scan rate of 50 mV for 10 cycles, as per described in [68]. Briefly, electrochemical cleaning helped to remove external contaminants and loosely attached structures on the surface of the SPCE, such as printing ink polymer residues and any other contaminants, to assure optimum performance, which can enhance the attachment of nanocomposites on the WE [57,69,70,71,72]. More overviews and details are illustrated in our previous work [68].

#### 2.3.2. Nanocomposite Preparation and Surface Modification

The MWCNT dispersion was prepared to produce a concentration of 0.05% wt. in isopropanol by sonication with 35% amplitude for 90 min. A biocompatible AuNP colloidal suspension was synthesized using the approach of photochemical-assisted synthesis with the help of branched poly (ethylene imine) (PEI, Mn = 60,000 g·mol^−1^) as a stabilizing agent. Thus, a colloidal solution suspension (0.03 M Au) of positively charged AuNPs was prepared [73,74].

The WE of the SPCE was firstly modified by evenly drop-casting a 3 µL of the MWCNT dispersion in 0.75 µL steps to ensure more even distribution of MWCNT on the electrode surface and to avoid concentric coffee-ring-like distribution [75,76]. The electrode was left to dry overnight and was followed by rinsing with DI water. After drying the electrode with N_2_ stream, 2 µL of the water-based AuNPs colloidal dispersion was drop-casted on the WE surface in 0.5 µL steps. The electrode was left to dry for hours, followed by rinsing with DI water and gently drying with the N_2_ stream.

#### 2.3.3. WE Preparation and HopQ-Ab Immobilization

**Figure 1:**
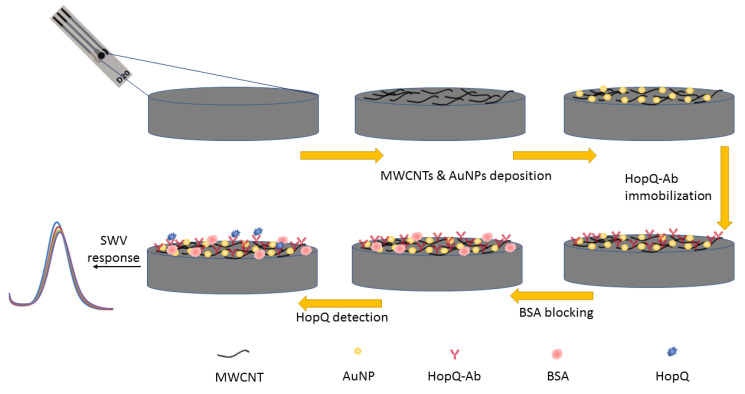
HopQ biosensor interface development process schematic diagram.

Regarding capture antibody immobilization, the MWCNT-COOH carboxyl group was activated with the versatile EDC/S-NHS chemistry. To achieve EDC/S-NHS activation, 5 µL of 4:1 molar ratio of the EDC/S-NHS solution in PBS buffer (10 mM, pH = 6) was dropped on the WE surface. The EDC/S-NHS was left on the WE for 1 h for conjugation in a dim atmosphere [77]. The S-NHS combined with -COOH activated by EDC and formed a semi-stable Sulfo-NHS ester, which had a half-life of hours in acidic mediums, that reacted with primary amines (-NH_2_) on the antibodies, forming covalently bonded antibodies to MWCNT-COOH [78]. As per the standard protocol, a 7.5 µL of HopQ-Ab (10 µg/mL, 10 mM PBS, pH = 7.4) was deposited on the WE surface and left for conjugation for 45 min [29,57,70,71,72,79]. The capture antibody (isoelectric point pH = 9.5) was positively charged at physiological pH = 7.4, which facilitated and enhanced the efficiency of immobilization due to attraction with the negative charge density on the MWCNTs [53,58,80,81,82]. To block non-specific binding sites, the WE were incubated with a 10 µL of 1% BSA in PBS (10 mM, pH = 7.4) for 120 min. Figure 1 depicts the HopQ biomarker-based electrochemical biosensor development process using HopQ-Ab immobilized on MWCNT/AuNP modified SPCE. The electrodes were rinsed with PBS and stored at 4 °C for further use and investigation.

### 2.4. Electrode Characterization

#### 2.4.1. Electrochemical Measurements

The sensor is characterized by CV and EIS measurements through the main development steps, such as deposition of nanomaterials, immobilization of HopQ-Ab and BSA, and detection of HopQ protein. CV was performed in the −0.6~+1.0 V range with a scan rate of 100 mV/s. A standard CV study for the fabricated sensor was performed at different scan rates from 10~100 mV/s. EIS was conducted in the frequency range from 0.1–10k Hz with 10 mV amplitude around the open circuit potential (OCP) voltage. All measurements were performed in 5 mM of K_3_[Fe(CN)_6_]/K_4_[Fe(CN)_6_] in 10 mM PBS solution with pH = 7.4 at room temperature.

#### 2.4.2. Surface Characterization by Scanning Electron Microscopy

SEM surface characterization was performed using FEI Nova NanoSEM 200 microscope to assess the surface morphology changes on the WE surface throughout MWCNT/AuNP and HopQ-Ab/BSA deposition. EDX spectroscopy of the WE surface was performed after MWCNT/AuNP deposition to identify the elements on the WE surface.

### 2.5. Analytical Performance and Detection of HopQ

#### 2.5.1. Detection of HopQ and Calibration Curve

Square wave voltammetry (SWV) measurements were performed in triplicates to evaluate HopQ detection performance. The parameters used were a voltage range from −0.3–+0.6 V with an amplitude of 0.1 V at 10 mV steps with 10 Hz frequency. All SWV measurements were performed using 5 mM of K_3_[Fe(CN)_6_]/K_4_[Fe(CN)_6_] in 10 mM PBS buffer solution with pH = 7.4 at room temperature. To obtain the calibration curve, the SWV peak current recorded for samples containing the analyte was subtracted from the peak current obtained for an analyte-free sample (control). This difference was then plotted against the logarithm of the analyte concentration expressed in ng/mL. Analytical performance analysis of artificial saliva spiked sample was performed with 10 ng/mL HopQ. The sensor was incubated in HopQ-spiked artificial saliva for 15 min, and the SWV response was recorded.

#### 2.5.2. Analytical Performance

The selectivity, cross-reactivity, reproducibility, and stability of the immunosensor were quantitatively evaluated. The selectivity analysis was performed by incubating the developed platform with buffer solutions containing comparable interferants such as BSA, Alpha-fetoprotein (Afp), and *H. pylori* CagA at excess concentrations.

The cross-reactivity was analyzed in solutions containing a mixture of HopQ at a concentration of 5 ng/mL and excess concentrations of interferants such as BSA, CagA, Afp, dopamine, and 17β-estradiol.

The reproducibility evaluation of the sensor was performed by independently preparing five copies of the sensor and recording the response for a solution with 5 ng/mL HopQ concentration. For the stability evaluation, the prepared platform was kept at 4 °C for 4 weeks while checking the difference in response between a blank and a 5 ng/mL sample every week.

## 3. Results

### 3.1. Electrochemical Measurements and Detection of HopQ

#### 3.1.1. Electrochemical Measurements

**Figure 2:**
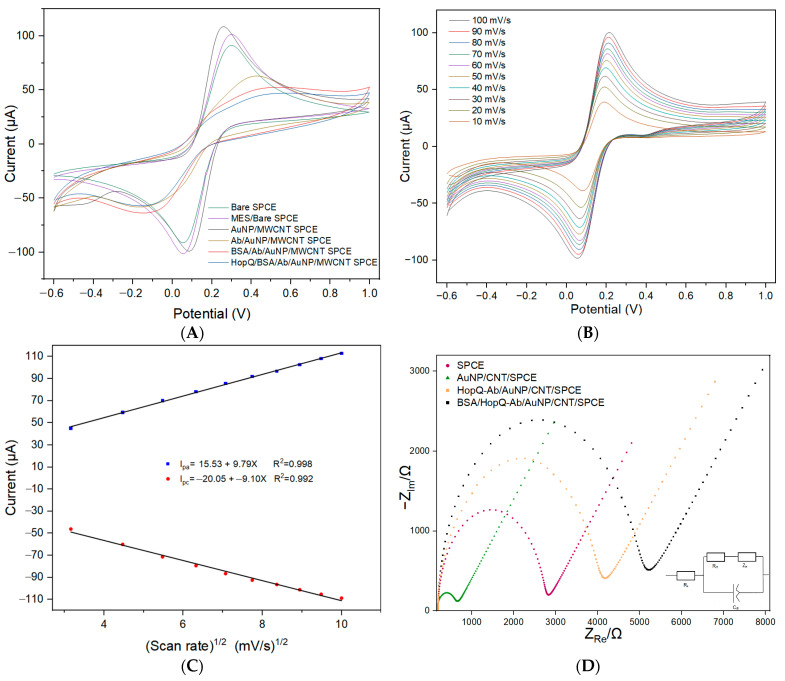
CV voltammogram studies for (**A**) bare SPCE electrode, pretreated SPCE electrode, AuNP/MWCNT SPCE electrode, HopQ-Ab/AuNP/MWCNT SPCE electrode, BSA/HopQ-Ab/AuNP/MWCNT SPCE electrode, and HopQ/BSA/HopQ-Ab/AuNP/MWCNT; (**B**) BSA/HopQ-Ab/MWCNT/AuNP SPCE at scan rate from 10 to 100 mV/s; (**C**) I_pa_ and I_pc_ of CV voltammograms at scan rates from 10 to 100 mV/s vs. $\sqrt{\mathrm{scan}\;\mathrm{rate}}$ curve with linear fitting; (**D**) Nyquist plot for frequency range 0.1–10k Hz with 10 mV amplitude around OCP.

The CV was performed along the electrode’s fabrication steps to confirm the sensing platform’s proof of concept. Figure 2A shows how the peak current of the CV voltammograms rises higher with surface modification using MWCNT and AuNP, with respect to a bare SPCE. Additionally, the CV peak separation voltage becomes narrower with nanocomposite modification, for which peak current and peak separation confirm faster charge transfer kinetics at the electrode surface which helps to increase sensor sensitivity and detection range [21]. By the immobilization of HopQ-Ab, the CV peak current was greatly diminished, and the voltage separation between the current’s peaks (ΔE_p_) considerably widened, implying slower charge transfer kinetics due to the added steric exclusion hindrance caused by the HopQ-Ab layer at the electrode surface. This surface blocking was further confirmed by adding BSA and an excess concentration of HopQ protein, I_pa_ and I_pc_ were further lowered, and ΔE_p_ became more dilated. This expected behavior supports the blocking effect of the bio-elements immobilized on the surface. These results also suggest that WE surface blocking results from the HopQ-Ab immobilization step.

The developed electrode performance was studied for several scan rates from 10–100 mV/s in the potential range from −0.6–+1.0 V in 10 mM PBS with a pH of 7.4, which contained 5 mM of K_3_[Fe(CN)_6_]/K_4_[Fe(CN)_6_] redox probe, as seen in Figure 2B. The voltammograms in Figure 2B notably demonstrate the increase in cathodic and anodic peak currents as the scan rate increases indicating a thinner diffusion layer at the electrode surface and faster diffusion charge transfer kinetics. Figure 2C shows that I_pa_ and I_pc_ are linearly related to the square root of the voltage scan rate, demonstrating the diffusion-controlled mechanism linearity on the electrode surface.

#### 3.1.2. Electrochemical Impedance Studies

**Figure 3:**
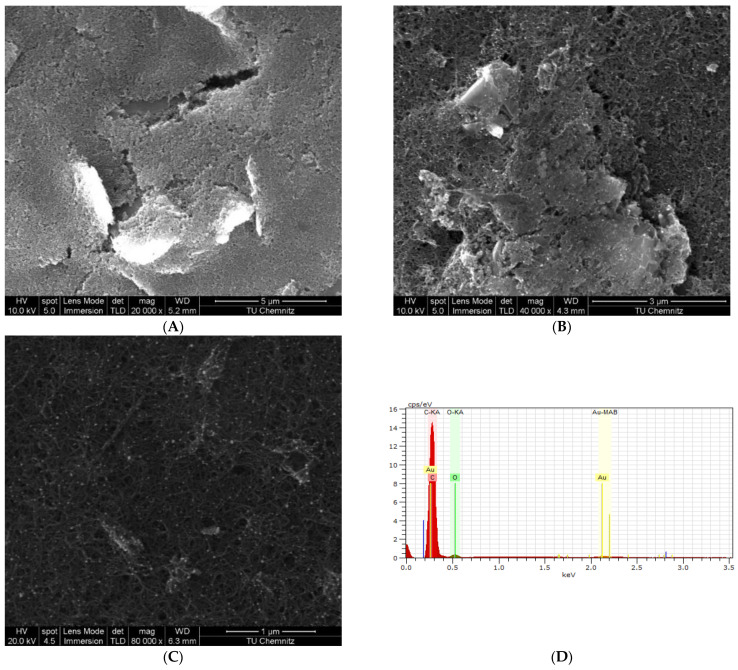
SEM micrograph image of bare SPCE electrode (**A**); AuNP/MWCNT/SPCE electrode (**B**); BSA/HopQ-Ab/AuNP/MWCNT/SPCE (**C**); EDX spectrum of AuNP/MWCNT/SPCE electrode (**D**).

EIS measurements were investigated to follow up on the electrode’s surface modifications, and the corresponding impedance spectrograms are presented in Figure 2D. EIS curves are fitted with Randle’s circuit model in Figure 2D. The fitting results, shown in Table 1, demonstrate the considerable drop in the charge transfer resistance (R_ct_) value after the deposition of nanoparticles on the WEs surface with respect to the bare graphite electrode. Additionally, the EDX results shown in Figure 3D confirm the deposition of MWCNT/AuNP on the WE surface. Oppositely, due to their weaker electrical activity, the R_ct_ value has increased approximately 11-fold upon immobilization of HopQ-Ab and the adsorption of BSA. This colossal increase in R_ct_ supports the evidence for successful immobilization and surface blocking. These conclusions are also supported by SEM spectroscopy in Figure 3A–C, in which a complete blockade of the surface is evident through the lower surface conductivity, even after increasing the incident beam intensity from 10 to 20 kV [83].

The value of CPE of the fitted EIS complex data indicates a considerable increase after the deposition of nanomaterials on the bare electrode, indicating a massive increase in surface area and more porous surface morphology. Nevertheless, the CPE value decreases after the immobilization of HopQ-Ab and blocking with BSA, indicating the additional spacing added by immobilizing BSA and HopQ-Ab [84].

### 3.2. Immunosensor Analytical Performance

**Figure 4:**
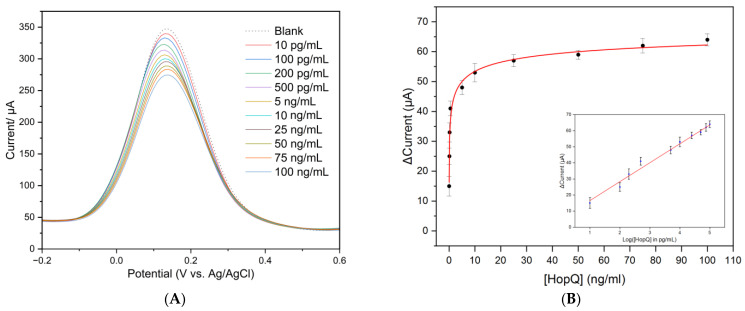
(**A**) SWV responses of BSA/HopQ-Ab/AuNP/MWCNT SPCE electrode with different HopQ concentrations, (**B**) variation in SWV peak current with respect to a blank solution vs. [HopQ] (and vs. Log[HopQ] for the inner plot). Error is the relative standard deviation for *n* = 3.

SWV technique was utilized to evaluate the sensing process performance of the immunosensor. Figure 4A exhibits the reduction in redox peak current vs. the concentration of HopQ protein. The study was carried out in 5 mM of K_3_[Fe(CN)_6_]/K_4_[Fe(CN)_6_] dissolved in 10 mM PBS solution with pH = 7.4 at room temperature. The measurement showed that peak current linearly decreased with increased HopQ concentration, mainly due to steric exclusion hindrance at the WE surface.

To quantify the immunosensor response, we plotted the variation of current intensity (I_0_ − I), where I_0_ is the current measured in a blank solution, and I is the current value obtained after each HopQ concentration. The resulting corresponding calibration curve is plotted in Figure 4B with equation y = 4.79455 + 11.77175 x, with R^2^ = 0.984. The sensitivity, calculated from the calibration curve’s slope, equals 11.77 µA/Log [HopQ, pg/mL]. The LOD of the designed immunosensor is calculated based on the criteria of the 3.3 times standard deviation of blank measurement for five electrodes according to equation (1a). LOD is calculated with the value of 2.0 pg/mL. The LOQ is calculated similarly based on the criteria of 10 times the standard deviation of blank measurements according to equation (1b), with a value of 8.6 pg/mL [43].

(1a) $$\mathrm{LOD}=10^{\frac{3.3\times \sigma }{a}}$$

(1b) $$\mathrm{LOQ}=10^{\frac{10\times \sigma }{a}}$$

where *σ* is the standard deviation of five electrodes blank measurement, and *a* is the slope of the calibration curve.

In the track of pathogen detection based on biomarkers, the sensor’s ability to detect the lowest possible concentrations of a biomarker is crucial and is a strong indicator of early detection. The obtained LOD of 2.0 pg/mL and LOQ of 8.6 pg/mL are the lowest reported in comparable research reports, as summarized in Table 2. LOD value particularly outstands the developed sensor from comparable work in means of detection at an earlier stage of infection. In addition, the linear range for the prepared electrode is 10 pg/mL–100 ng/mL, which also outperforms detection platforms reported in the state-of-the-art, Table 2. The incorporated HopQ as a biomarker into the label-free immunosensor is inspired by the fact that *H. pylori* utilize HopQ to attach to the epithelial layer and transfer its virulence factors, such as CagA, BabA, and VacA, which is one of the earliest steps of infection [8]. Relying on virulent factors as biomarkers has merit but might not indicate the earliest possible stages of infection, especially when the LOD is higher than the reported in this work, as seen in Table 2. However, HopQ is an outer membrane protein (OMP) and naturally exists even before active infection starts or even for in vitro detection, such as in food or drinking water samples. This makes HopQ a novel selection as a reliable *H. pylori* biomarker. The fact that HopQ is an OMP inspired us to utilize it in a simpler detection approach by targeting more non-invasive body fluids, such as saliva, that do not require complicated sample collection or preparation. The use of saliva can waive the urge to rely on body excretions or fluids that require rigorous collection and preparation, such as serum or excrement. SPCE modified with MWCNT/AuNP/HopQ-Ab/BSA is cost-effective and simple to fabricate, requiring minimal sample preparation for measurement compared to alternative approaches with an immunosensing approach. The nanocomposites of MWCNT/AuNP synergically serve as excellent surface modifiers that lay out a platform for the covalent immobilization and stabilization of capture antibody and effectively enhance sensor sensitivity due to its high catalytic effectiveness. Therefore, the developed sensor represents one step toward a promising effective, simple, and point-of-care compatible tool that eliminates the need for complicated sample collection and preparation in clinical practices and broadens the patient range.

In the context of antibody–antigen interactions, Hill’s model describes the dissociation constant (*K_d_*) of the antibody–antigen complex. The dissociation constant measures the strength of the interaction between the antibody and antigen, with a lower value indicating a stronger interaction. It is one of the most important factors in the immunorecognition process in immunosensors. It offers information on the affinity between the antibody immobilized on the sensor’s surface and the protein (antigen). In this work, the *K_d_* value is calculated by fitting the experimental data using Hill’s model according to the below equation:

(2) $$S\left(C\right)=\frac{A\times C^{\alpha }}{K_{d}^{\alpha }+C^{\alpha }}$$

where *S*(*C*) is the normalized current variation (I_0_ − I)/I_0_ as a function of concentration, *α* is Hill’s coefficient, *A* is an empirical constant, and *K_d_* is the dissociation constant. Fitting the measurement data according to Hill’s model, the dissociation constant is $K_{d}=4.605\times 10^{-10}$ mg/mL. The value range of the reported $K_{d}$ is very low, indicating a solid attachment in this recognition system, which is expected for antibody–antigen immunoreaction [85].

### 3.3. Selectivity and Cross-Reactivity

The selectivity and cross-reactivity of immunosensors are crucial to assess the analytical performance of the biosensor in complex samples such as saliva, blood serum, and excrement. In this dimension, the selectivity and cross-reactivity evaluation of the fabricated sensing platform was studied in the presence of an excess concentration of various interferants (10 times the highest concentration in the detection range). Several interferants, such as CagA, AFP, BSA, dopamine, and estrogen, have been used.

#### 3.3.1. Selectivity

**Figure 5:**
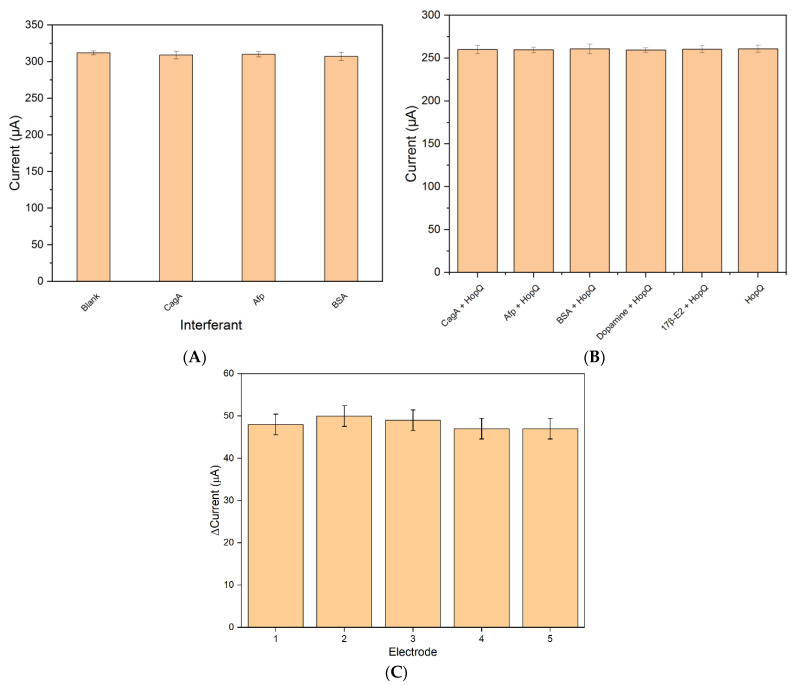
(**A**) Interferent study of BSA/HopQ-Ab/AuNP/MWCNT SPCE electrode with 5 ng/mL interferant concentration. (**B**) Interferent study of BSA/HopQ-Ab/AuNP/MWCNT SPCE electrode with 5 ng/mL HopQ antigen. (**C**) SWV peak current response of identically fabricated electrodes with the same criteria; the error bar is for *n* = 5.

The selectivity of the platform was studied against CagA because it is an *H. pylori* protein, Afp because it is a human protein, and BSA, which has been used in electrode fabrication. The peak of the SWV after incubation at 15 min did not return any significant difference in the current response from a blank solution for this selectivity test, Figure 5A.

#### 3.3.2. Cross-Reactivity

The cross-reactivity investigation was conducted in the presence of 5 ng/mL HopQ protein. Figure 5B shows the cross-reactivity study of the fabricated immunosensor platform incubated in a solution of 5 ng/mL HopQ and excess concentrations of different interferents. The results show that a maximum of 14% variation in current was observed for the BSA as interferant in the presence of HopQ. No significant changes are noticed in the SWV peak currents compared to the one measurement of HopQ only. Therefore, the developed platform showed excellent specificity.

### 3.4. Reproducibility

One of the most important concerns in the field of sensors is sensor-to-sensor reproducibility. In this regard, the reproducibility of the sensing platform has been studied by independently preparing five electrodes. The reproducibility was observed by comparing the performance of the equally prepared five electrodes with 5 ng/mL HopQ concentration. Figure 5C depicts the current response of the five different electrodes, and it is evident that it varied slightly (RSD = 2.42%), which indicates excellent reproducibility of the immunosensor.

### 3.5. Shelf-Life Studies and Comparison with Other Platforms

Sensors with biological elements included in their fabrication are considered fragile and prone to be less resilient upon long-term storage. In this dimension, the shelf-life stability of the prepared immunosensor stored at 4 °C was investigated by checking the peak current every week in a 5 ng/mL of HopQ solution. The SWV peak current difference was reduced by only 15% after 4 weeks, which suggests minimal degradation occurred to the developed interface surface.

### 3.6. Artificial Saliva Samples

Since we aimed to detect HopQ in a complex sample matrix such as saliva, the developed sensing platform was tested with a HopQ-spiked artificial saliva sample. The recovery percentage of 10 ng/mL of HopQ-spiked artificial saliva triplicate was calculated using the standard method. The results demonstrated a 107.6% recovery of the expected peak current with RSD = 3.18%, which supports the developed biosensor’s accuracy and usability for saliva real sample analysis in the future.

## 4. Conclusions

This work discusses the successful development of a novel electrochemical *H. pylori* immunosensor utilizing HopQ as a biomarker with polyester substrate-based SPCE modified with MWCNTs and decorated with AuNPs. The developed sensor is characterized by electrochemical techniques (CV, EIS, SWV) and spectroscopy (SEM, EDX) where the results confirm the sensor’s fabrication steps and the interpretation of surface properties were obtained. The detection analytical performance of BSA/HopQ-Ab/AuNP/CNT/SPCE immunosensor is investigated by SWV, revealing a wide linear range of HopQ detection of 0.01–100 ng/mL with excellent linearity, low LOD of 0.002 ng/mL, 0.008 ng/mL LOQ, and with a conjugation time of only 15 min. Moreover, the immunosensor is very specific and selective, with 107.6% recovery in spiked artificial saliva samples with RSD = 3.18%. The immunosensor’s output maintains 85% of its activity after 4 weeks. The sensor’s selectivity and cross-reactivity studies exhibit excellent HopQ selectivity of the biosensor with a negligible variation of the peak current with respect to blank measurement, with less than 15% variation in current in the presence of HopQ/interferants mixture. The reproducibility of the biosensor is RSD = 2.42 for *n* = 5.

The utilization of MWCNT/AuNP nanocomposite allows the capture antibody covalent bonding and boosts SPCE performance. It is promising in realizing cost-effective biosensors compatible with point-of-care test technology for *H. pylori* detection to curb its impact on humanity. It is worth noting that this work is the first to report the utilization of HopQ as a biomarker for *H. pylori* in an electrochemical immunosensor and the first to report the dissociation constant of HopQ/HopQ-Ab interaction, to the best of the author’s knowledge.

Reliable detection of *H. pylori* at very low concentrations still embraces several challenges related to sensing setups, such as minimizing sample collection and preparation, test time, and minimizing the need for highly skilled staff and sophisticated equipment. The findings in this work suggest a high potential of biosensors for reliable detection of *H. pylori* in saliva, providing a cost-effective, simpler, and reliable alternative to blood serum, stool samples, or urea breath tests, which broadens the patient test target group.

## Figures and Tables

**Table 1:** Randle’s equivalent circuit parameter fittings for the simplified equivalent circuit of the Nyquist plot data are presented in Figure 2D.

Electrode	R_s_ (Ω)	R_ct_ (Ω)	CPE (F)
SPCE	192.2	2306	6.24 × 10^−7^
AuNP/CNT/SPCE	165.4	416.8	3.79 × 10^−6^
HopQ-Ab/AuNP/CNT/SPCE	194.05	3917.3	1.13 × 10^−6^
BSA/HopQ-Ab/AuNP/CNT/SPCE	197.7	4720	9.22 × 10^−7^

**Table 2:** Limit of detection, detection range, and stability properties for different studies [61,62,63,64,65].

Ref.	Performance of Reported *H. pylori* Sensors						
	Interface	Detection Method	Biomarker	LOD (ng/mL)	Linear Range (ng/mL)	Stability at 4 °C (Weeks)	
[64]	CagA-Ab/ZnO*-T/SP-AuE	DPV	CagA	0.2	0.2–50	8–9	Up to 90%
[62]	CagA-Ab/TiO_2_-NPs/c-MWNCT/Pin5COOH/AuE	SWV	CagA	0.1	0.1–8.0	~3	90%
						~6	50%
[61]	CagA-Ab/Pt_nano_/PEDOT/rGO/AuE	EIS	CagA	0.1	0.1–30	~8	60–70%
[65]	BabA-Ab/Pd_nano_/rGO/PEDOT/AuE	EIS	BabA	0.2	0.2–20	8–9	70%
[63]	VacA-Ab/g-C_3_N_4_/ZnO/AuE	DPV	VacA	0.1	0.1–12.8	~2	94%
This work	BSA/HopQ-Ab/AuNP/CNT/SPCE	SWV	HopQ	0.002	0.01–100	4	~85%
						8	~60%

ZnO*-T: Irradiated Zinc Oxide Tetrapods, SP-AuE: screen printed gold electrode, AuE: gold electrode, TiO_2_-NPs: Titanium oxide nanoparticles, c-MWCNT: carboxylated multi-walled carbon nanotubes, Pin5COOH: polyindole carboxylic acid, Pd/Pt_nano_: palladium/platinum nanoparticles, PEDOT: poly(3,4-ethylenedioxythiophene), rGO: reduced graphene oxide, g-C_3_N_4_: graphitic carbon nitride.

## Data Availability

Not applicable.
